# Very Severe Aplastic Anemia in a 26-Year-Old Male: Implications for Prognosis and Treatment Options

**DOI:** 10.7759/cureus.45750

**Published:** 2023-09-22

**Authors:** Ahmed Alobaidi, Ahmed Albadry, Anne Murray

**Affiliations:** 1 Internal Medicine, Methodist Health System, Dallas, USA; 2 Faculty of Medicine, Charles University in Prague, Prague, CZE; 3 Clinical Research Institute, Methodist Health System, Dallas, USA

**Keywords:** pancytopenia, hematopoietic stem cell transplant, failure of the bone marrow, neutropenic fever, very severe aplastic anemia

## Abstract

Aplastic anemia (AA) is a hematopoietic stem cell (HSC) disorder characterized by the loss of HSCs, bone marrow failure, and peripheral pancytopenia. AA is classified as very severe (VSAA), severe (SAA), or non-severe (NSAA) based on the severity criteria. This classification system has implications for the prognosis and treatment options offered to patients. The prognosis of AA has improved over the past several decades with the advancements in supportive care, HSC transplant (HCT), and immunosuppressive therapy (IST). In this report, we present the case of a 26-year-old male diagnosed with VSAA after presenting with severe neutropenia and fever. The patient ultimately underwent HSC transplantation.

## Introduction

Aplastic anemia (AA) is a life-threatening hematologic disorder in which all peripheral blood cell lines are depressed due to bone marrow failure. Anemia, bleeding, and infections are the usual manifestations of AA. AA is a rare entity, with an annual incidence of <2.5/million people in the United States [1], and it almost equally affects both genders. The age groups most commonly associated with AA are 10-25 years and >60 years, although it can occur at any age [2]. AA can be inherited or acquired. Acquired AA accounts for about 70% of all cases [3], while inherited AA represents >25% of AA in pediatric populations and 5-15% of AA in adults <40 years of age. The incidence of inherited AA is on the rise due to its increased recognition and improved access to testing for multiple genetic syndromes [3].

The mechanisms underlying AA pathogenesis resulting in progenitor cell damage and ultimately bone marrow failure and pancytopenia are very complex. Changes in immune function in AA are linked to autoimmune destruction of hematopoietic stem cells (HSCs) by abnormally activated T lymphocytes and associated lymphokines, reduced natural killer cell surveillance function, and reduced immune tolerance of dendritic cells [2,4]. Other implicated mechanisms include genetic changes in HSCs causing severe and consistent deficiencies in HSC number and function. Such genetic changes can result from external factors such as viruses (e.g., non-A viral hepatitis, Epstein-Barr virus, and HIV), radiation, and chemotherapeutic drugs [2]. Also, high expression of Fas antigen can induce apoptosis in CD34 progenitor cells resulting in HSC depletion [5,6].

AA can result in significant mortality and morbidity due to infections associated with severe neutropenia and life-threatening bleeding from thrombocytopenia. While AA without HSC transplant (HCT) carries a mortality rate as high as 70% in two years [3], the prognosis can improve drastically to an 80-90% 10-year survival rate in young, healthy individuals with matched related donors if HCT is performed early.

## Case presentation

A previously healthy 26-year-old male patient presented with fever and dyspnea for two weeks. His fever was intermittent and his temperature had been as high as 40.6 °C at home. The fevers were associated with generalized weakness, fatigue, malaise, dyspnea on exertion, and a significant reduction in exercise tolerance. His wife had noted new “spots” on both of his legs and chest. The patient reported a recent history of a “gum infection” with persistent gum bleeding following tooth brushing. He had been treated with an unknown oral antibiotic, oral chlorhexidine mouthwash, and ibuprofen, but this condition had persisted. He denied having night sweats, sick contacts, weight loss, hemoptysis, chest pain, cough, nausea, vomiting, or recent travel outside the state of Texas. He had presented to urgent care twice for his complaints and had been given intramuscular injections of ceftriaxone for reported pneumonia.

On physical examination, he was febrile with a temperature of 39.5 °C and tachycardic with a heart rate of 121 beats per minute. Otherwise, his vital signs were within normal limits. The physical examination was remarkable for a pale-appearing and diaphoretic male with non-blanching petechiae over the chest, abdomen, and both legs and a soft, non-tender abdomen without palpable liver or spleen. No palpable lymph nodes were noted in the cervical, axillary, or inguinal regions. The laboratory data was significant for the following: white blood cell count of 1.0 x 10^3^/µL (differential: 10.8% neutrophils, 84.2% lymphocytes, and 5% monocytes), absolute neutrophil count of 108 neutrophils/µL, hemoglobin of 6.7 g/dL, hematocrit of 19.2%, reticulocyte count of 14.7 x 10^9^/µL (0.53%), platelet count of 6,000 platelets/µL, INR of 1.2, ferritin of 620 µg/L, iron saturation of 75%, total iron-binding capacity of 195 µg/dL, folate of 7.2 ng/mL, vitamin B12 of 578 pg/mL and thyroid stimulating hormone of 3.0 mIU/mL. Hep A/B/C and HIV serologies were negative, and blood cultures and a respiratory virus panel were also negative.

**Figure 1:**
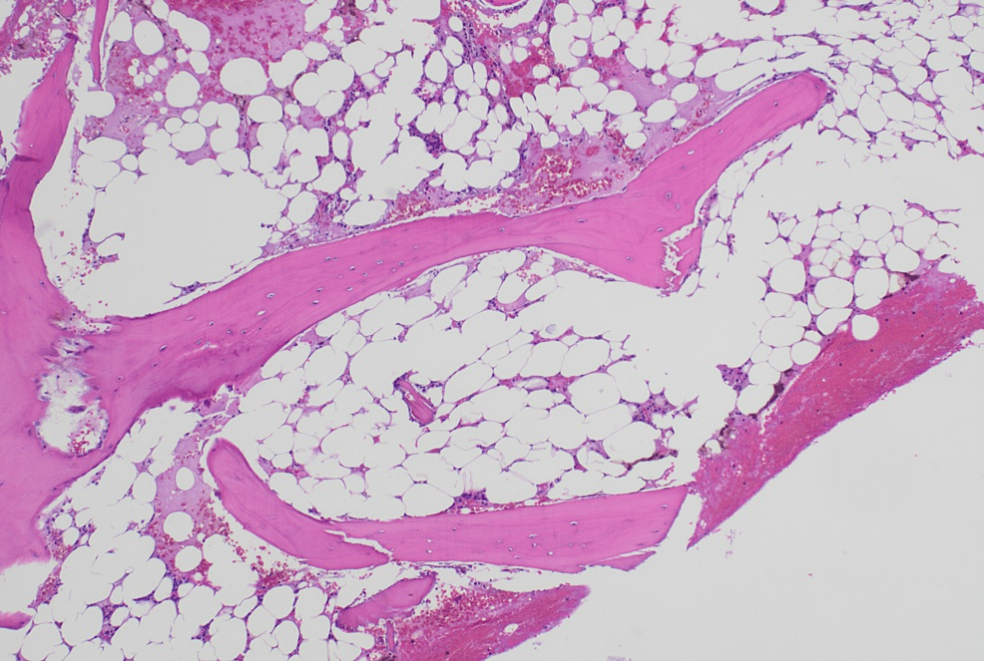
Hematoxylin and eosin stain of bone marrow biopsy The image shows a hypocellular bone marrow with <5% cellularity

The patient's liver function tests and a basic metabolic panel were normal, and urinalysis and chest X-ray were unremarkable. Venous and Doppler ultrasound of the abdominal organs showed a normal-sized spleen with patent splenic, portal, and hepatic veins. A peripheral blood smear showed pancytopenia without abnormal cells or blasts. A bone marrow biopsy showed marked decreased cellularity at 5% with significant fatty infiltration noted; no dysplastic changes or abnormal cells or blasts were seen (Figure 1). Cytogenetics and fluorescence in situ hybridization showed a normal 46,XY karyotype with no abnormal clones detected. Concurrent flow cytometry of the bone marrow and peripheral blood demonstrated no immunophenotypic evidence of a hematolymphoid malignancy.

On admission, the patient was given IV fluids, platelet and red blood cell transfusions, and empirical antibiotics (vancomycin and cefepime) for the treatment of sepsis of an unknown source. His severe pancytopenia was later determined to be from AA. His fever resolved 24-48 hours after admission. A hematology consultation recommended a bone marrow transplant evaluation, and hence the patient was transferred to a transplant center. He was given immunosuppressive therapy (IST) with anti-thymocyte globulin (ATG) and cyclosporine A (CSA) followed by an HCT from his HLA-matched brother. It has been three years since he underwent the transplant, and the patient is currently in stable health without major complications from the transplant or any evidence of graft failure.

## Discussion

Among the numerous causes of pancytopenia, hypoplastic/aplastic etiologies are the leading ones in Western countries [7]; the diagnosis in this case was AA. Evaluation of a patient with suspected AA should initially focus on the exclusion of other causes of peripheral pancytopenia in the presence of bone marrow failure, as demonstrated on a bone marrow biopsy. Also, the presence of a coexisting disorder, such as paroxysmal nocturnal hemoglobinuria (PNH), myelodysplastic syndrome (MDS), or acute leukemia should be ruled out.

Hypoplastic MDS represents 10-15% of MDS cases and must be differentiated from AA as many features of the two disorders overlap, including pancytopenia in the peripheral blood and hypocellularity of the bone marrow [8]. Generally, MDS can be differentiated from AA by the presence of dysplastic changes in the bone marrow, ringed sideroblasts on a bone marrow biopsy, and the presence of molecular and karyotypic abnormalities, which were lacking in our patient. The differentiation of hypoplastic MDS from AA can be challenging because some dysplastic changes in MDS can be subtle [9]. PNH can be differentiated from AA generally by the presence of hemolytic anemia and flow cytometry markers typical of PNH. A bone marrow biopsy typically shows hypercellular bone marrow with PNH. Infiltrative disorders causing bone marrow failure including metastatic solid tumors and infectious agents also need to be excluded to establish a definitive diagnosis of AA. Bone marrow biopsy in our patient did not show evidence of leukemia. Bone marrow suppression and associated peripheral pancytopenia can mimic AA; however, our patient did not have a history of exposure to cytotoxic medications, radiation, or common offenders (e.g., antiepileptic medications). A notable feature in drug-induced bone marrow suppression is reversibility after an offending agent is discontinued. Hypersplenism can also be associated with severe pancytopenia; however, our patient’s spleen size appeared to be normal on ultrasound.

AA severity is determined by neutrophil count: those with a count of 0-0.2, 0.21-0.5, or >0.5 x 10^9^ are designated as very severe (VSAA), severe (SAA), or non-severe (NSAA) AA, respectively [2]. This classification is important in terms of patient selection for therapy and also carries prognostic implications. Patients who underwent bone marrow transplants from matched siblings exhibited a 10-year survival rate of 82%, 72%, and 53% for those aged 1-20 years, 21-40 years, and >40 years, respectively [10]. Given this finding, it is recommended that patients with SAA or VSAA who are medically fit and <40 years of age undergo HCT with a related available HLA-matched donor [11,12]. IST, including ATG and CSA, is generally a better option for older patients with SAA or those with underlying medical conditions (i.e., those at higher risk of complications with HCT).

The hematopoietic response rate after ATG/CSA has been reported to be 60-70% and the probability of survival at five years is 60-85% [11]. The clinical course of NSAA is variable and does not require therapy in many instances [11]. A significant proportion of NSAA patients will stabilize or experience remission, while 20-33% can progress to SAA and might ultimately require definitive therapy with HCT or IST [13]. Survival in patients with AA has improved markedly over the past several decades due to advances in HCT, immunosuppressive medications, biologic agents, and supportive care [14]. The two-year mortality rate for patients with SAA or VSAA who receive supportive care alone approaches 80%, with invasive fungal infections and bacterial sepsis being the most frequent causes of death [11]. However, with advances in HCT and IST, the 10-year survival rate has reached as high as 80-90% [15-17].

## Conclusions

HCT therapy for younger AA patients with an HLA-matched donor is considered the first line of therapy, with IST being reserved for older patients and those with underlying medical conditions that render them high-risk for HCT. The severity of AA and patient age are among the most important factors determining the prognosis of AA and response to therapy and transplant. The survival rates of AA patients have improved over the past several decades thanks to advances in therapies and supportive care.
